# Probiotic *Bifidobacterium breve* Prevents Memory Impairment Through the Reduction of Both Amyloid-β Production and Microglia Activation in APP Knock-In Mouse^1^

**DOI:** 10.3233/JAD-215025

**Published:** 2022-02-15

**Authors:** Mona Abdelhamid, Chunyu Zhou, Kazuya Ohno, Tetsuya Kuhara, Ferdous Taslima, Mohammad Abdullah, Cha-Gyun Jung, Makoto Michikawa

**Affiliations:** a Department of Biochemistry, Graduate School of Medical Sciences, Nagoya City University, Nagoya, Japan; b Next Generation Science Institute, Morinaga Milk Industry Co., Ltd., Zama, Japan; c Department of Neuroscience, Mayo Clinic, Jacksonville, FL, USA

**Keywords:** ADAM10, Alzheimer’s disease, amyloid-β production, *Bifidobacterium breve* MCC1274, ERK, glial activation, HIF-1*α*, novel object recognition, synapses

## Abstract

**Background:**

Probiotic supplementation reestablishes microbiome diversity and improves brain function in Alzheimer’s disease (AD); their molecular mechanisms, however, have not yet been fully illustrated.

**Objective:**

We investigated the effects of orally supplemented *Bifidobacterium breve* MCC1274 on cognitive function and AD-like pathologies in *App^*NL*-*G*-*F*^* mice.

**Methods:**

Three-month-old *App^*NL*-*G*-*F*^* mice were orally supplemented with *B. breve* MCC1274 for four months. The short-term memory function was evaluated using a novel object recognition test. Amyloid plaques, amyloid-β (Aβ) levels, Aβ fibril, amyloid-β protein precursor and its processing enzymes, its metabolic products, glial activity, and cell proliferation in the subgranular zone of the dentate gyrus were evaluated by immunohistochemistry, Aβ ELISA, western blotting, and immunofluorescence staining. The mRNA expression levels of pro- and anti-inflammatory cytokines were determined by qRT-PCR analysis.

**Results:**

We found that the oral *B. breve* MCC1 274 supplementation prevented memory impairment in *App^*NL*-*G*-*F*^* mice and decreased hippocampal Aβ levels through the enhancement of the a-disintegrin and metalloproteinase 10 (ADAM10) level. Moreover, administration of the probiotic activated the ERK/HIF-1*α* signaling pathway responsible for increasing the ADAM10 level and also attenuated microglial activation, which in turn led to reduction in the mRNA expression levels of pro-inflammatory cytokines in the brain. In addition, *B. breve* MCC1274 supplementation increased the level of synaptic proteins in the hippocampus.

**Conclusion:**

Our findings support the possibility that oral *B. breve* MCC1274 supplementation might be used as a potential preventive therapy for AD progression.

## INTRODUCTION

Alzheimer’s disease (AD) is a neurodegenerative disease, accounting for 50% of all dementia cases in the world [1]. Senile plaques, formed from amyloid-β (Aβ) deposition, and neurofibrillary tangles, due to the accumulation of the abnormally tau hyperphosphorylation, are the pathological hallmarks of AD. Aβ is produced following cleavage of the amyloid-β protein precursor (AβPP) by β- and *γ*-secretases [2]. Alternatively, it can undergo non-amyloidogenic processing through the *α*-site AβPP-cleaving enzyme (*α*-secretase), including a-disintegrin and metalloproteinase 10 (ADAM10), in the middle of the Aβ sequence, thereby precluding Aβ production [3]. Therefore, the inhibition of Aβ production and deposition is still considered one of the promising AD therapeutic approaches. Other pathogeneses of AD involve neuroinflammation, deficits in energy metabolism, and vascular degeneration [4–7]. However, current therapeutic strategies based on existing theories have not yet yielded any benefits; thus, there is an urgent need for new treatment strategies for AD.

Recently, the microbiome-gut-brain axis has been the focus of clinical research studies. The connection between the gut and the brain is achieved by one of these three pathways: direct link from the gastrointestinal tract to the intrinsic nervous system, inflammatory cytokines released by microorganisms, which modulate systemic immunity, and hormonal signaling by the hypothalamic-pituitary-adrenal axis [8–10]. Probiotics are living microorganisms that have health benefits and contribute to the immune and digestive systems [11]. Reduction of microbiota diversity can cause cerebrovascular degeneration, inflammation, tau pathology, and Aβ accumulation [12–14]. Moreover, the low abundance of anti-inflammatory bacteria (*Bifidobacterium*) and the rising of proinflammatory bacteria (Proteobacteria) have been associated with AD [15]. Gut microbiota secretes some bacterial neurotransmitters; for example, *Bifidobacterium* species secrete gamma-aminobutyric acid (GABA) [16], which could regulate brain functions. The gut microbiome also produces some beneficial metabolites such as propionic, valeric, and butyric acids, which can inhibit inflammation and Aβ accumulation [17]. It has been reported that probiotics supplementation improves brain performance and alters the gut microbiome environment, suggesting that probiotics supplementation may be a potential strategy to delay AD progression.

It has been reported that microbiota impairment inhibits microglial activation and its phagocytic activity of Aβ [18]. Neuroinflammation mediated by microglia plays a critical role in AD pathogenesis. Microglia have dual functions, neuroprotective or neurotoxic effects. Activation of microglia under physiological conditions enhances Aβ phagocytosis and clearance leading to reduced Aβ accumulation. On the other hand, chronically activated microglia cause neuronal loss owing to their involvement in the release of proinflammatory cytokines such as IL-1β and IL-6 [19, 20], which have been shown to be increased in the brains of AD patients and AD mouse models [21, 22].

Our previous study demonstrated that oral *Bifidobacterium breve* strain A1 (*B. breve* MCC1274; synonym, *B. breve* A1) supplementation rescued cognitive deficits in ddY mice induced by intracerebroventricular injection with Aβ, through the suppression of hippocampal inflammation and immunoreactive genes [15]. Furthermore, our recent study demonstrated that oral *B. breve* MCC1274 supplementation for 16 weeks enhanced the cognitive functions of subjects with mild cognitive impairment (MCI) [23]. These findings suggest that oral *B. breve* MCC1274 supplementation can be a potentially effective treatment for AD. However, the molecular mechanism(s) by which this probiotic improves cognitive function remains unknown. Since ddY mice do not show AD phenotypic features, such as Aβ deposition, we could not investigate whether the oral *B. breve* MCC1274 supplementation is associated with Aβ production and deposition and glial activation in the brain. Therefore, to understand the mechanisms of action and explore the possibility of this probiotic pharmaceutical potential, this study was performed using *App^*NL*-*G*-*F*^* as a mouse that shows the phenotypic features of AD [24]. In this study, we explored the effects of the oral *B. breve* MCC1274 supplementation on cognitive function and pathogenesis of AD, including its effects on Aβ production, synaptic protein levels, and glial activation, and to elucidate the mechanism by which probiotic supplementation improves cognitive function. As predicted from our previous studies, oral supplementation of this probiotic attenuated cognitive impairment in *App^*NL*-*G*-*F*^* mice. Furthermore, we found for the first time that oral *B. breve* MCC1274 supplementation reduced Aβ production by increasing the ADAM10 level in the hippocampus, and also attenuated microgliosis accompanied by the downregulation in pro-inflammatory cytokines in the brain. This study as well as our previous studies’ findings suggest that oral *B. breve* MCC1274 supplementation might be used as a potential preventive therapy for AD progression.

## MATERIALS AND METHODS

### Preparation of probiotic

*B. breve* MCC1274 (stocked in Morinaga Culture Collection, Zama, Japan; synonym, *B. breve* A1) was provided by Morinaga Milk Industry Co., Ltd. (Zama, Japan) and prepared as described in our previous study [15]. Briefly, *B. breve* MCC1274 was isolated from infant feces. It was then collected by centrifugation after growing on a medium rich in yeast and glucose, then lyophilized and stored at 4°C until use. Before oral administration to experimental mice, *B. breve* MCC1274 cells were suspended in saline at a concentration of 1×10^9^ cfu/200*μ*l/day/mouse.

### Animal and probiotic treatments

**Fig. 1:**
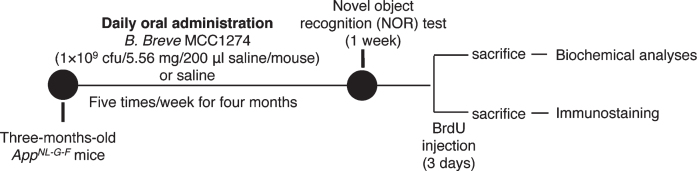
Experimental procedure. Three-month-old *App^*NL*-*G*-*F*^* mice were assigned randomly into the vehicle and probiotic groups: the vehicle group (*n* = 26) received saline and the probiotic group (*n* = 26) was supplemented with *B. breve* MCC1274 (1×10^9^ cfu/5.56 mg/200*μ*l saline/mouse) via oral gavage five times/week for four months. At the end of supplementation, feces were collected, and then the mice were behaviorally evaluated by novel object recognition (NOR) test or injected with BrdU. The mice were sacrificed and brains collected for biochemical analyses and immunostaining.

App knock-in (KI) mice (*App^*NL*-*G*-*F*^*), which overproduce Aβ without overexpressing AβPP, with the Swedish, Iberian, and Arctic mutations, were obtained from RIKEN Center for Brain Science (Wako, Japan). These mice show Aβ deposition starting at the age of two months until saturation at the age of seven months and exhibit memory impairment beginning at six months [24]. Three-month-old *App^*NL*-*G*-*F*^* mice were assigned randomly into vehicle and probiotic groups: the vehicle group (*n* = 26) received saline and the probiotic group (*n* = 26) was supplemented with *B. breve* MCC1274 (1×10^9^ cfu/5.56 mg/200*μ*l saline/mouse) via oral gavage five times/week for four months. The mice were housed on 12-h light and dark cycle provided with water ad libitum and oriental basal diet (Oriental Yeast Co., Tokyo, Japan). All the experiments were carried out in conformity with the National Institute of Health Guide for the Care and Use of Laboratory Animals and were approved by Nagoya City University Institutional Care and Use of Laboratory Animals committee. The experimental design is shown in Fig. 1.

### Novel object recognition test

After the probiotic supplementation for four months, the memory of the mice was tested using the novel object recognition (NOR) test. The NOR test is a learning and memory test to evaluate visual recognition, which is based on the principle that animals tend to explore new objects. The NOR test was carried out as reported previously [25, 26]. Briefly, it consists of three stages: a habituation stage, a training stage, and a testing stage. In the habituation stage, the mouse was allowed to explore a box without any objects for 3 min for three days. During the training stage, the mouse explored the box for 5 min, which contained two familiar objects. The testing stage was 24 h after the training stage. In this stage, the box contained a new object (novel) and a familiar object, and the mouse was let free to explore both objects for 5 min. A video camera was used to record the exploration time in both training and testing stages. During the experiments, the objects were matched in terms of their emotional neutrality and physical complexity of the mice. A discrimination index (DI) reflects the duration of exploration for the novel object compared to the familiar object as a proportion of the total exploration time of the mouse for both objects. Exploration is defined as touching or sniffing the object. NOR analysis was performed blinded.

### Aβ ELISA

Brain Aβ levels were determined as described in a previous report [27]. Hippocampal and cortical tissues were homogenized and centrifuged at 100,000 rpm for 20 min. The supernatants were used for soluble AβPP (sAβPP) and soluble Aβ measurements. The pellets were dissolved in 10 volumes of 6 M guanidine hydrochloride, sonicated, and incubated for 1 h at room temperature, then centrifuged at 100,000 rpm for 20 min, which were used for insoluble Aβ determination. Aβ_40_ and Aβ_42_ were determined using ELISA kits (294-64701, 290-62601 respectively, Wako Pure Chemical Industries, Osaka, Japan). The levels of Aβ were normalized to the weight of the brains.

### Western blotting

Hippocampal and cortical tissues were homogenized in lysis buffer containing a protease inhibitor mixture. Equal protein amounts were subjected to SDS-PAGE and immunoblotting on a membrane (IPVH00010, Millipore). This was followed by blocking and incubation with primary antibodies: anti-ADAM10 (1 : 1000, MAB19026, Millipore), anti-APP (1 : 1000, MAB348, Millipore), anti-PS1 (1 : 1000, MAB5232, Millipore), anti-BACE1 (1 : 1000, MAB931, R&D), anti- sAβPPβ (1 : 1000, #10321, IBL), anti-6E10 (1 : 1000, SIG-39300,Vovanc), anti-PSD95 (1 : 1000, #9900, Cell signaling), anti-SYT (1 : 1000, #612714, BD Transduction), anti-HIF-1*α* (1 : 1000, #3716, Cell signaling), anti-PKC (1 : 1000, 610108, BD Transduction), anti-phospho-PKC (1 : 1000, #06-822, Upstate Cell Signaling), anti-ERK (1 : 1000, #9102, Cell Signaling), anti-phospho-ERK (1 : 1000, #9101, Cell Signaling), anti-GFAP (1 : 1000, G3893, Sigma), anti-total tau (1 : 1000, 806401, Biolegend), anti-phospho-tau (AT180, 1 : 1000, MN1040, Invitrogen), anti-phospho-tau (PHF1, 1 : 1000, MN1020, ThermoFisher scientific), anti-Iba1 (1 : 1000, 019-19741, Wako), and anti-actin (Proteintech Group). Washing and incubation with a proper HRP-conjugated secondary antibody was the done. Immuno Star Zeta or Immuno Star LD (295-72404, 290-69904 respectively, Wako) was used for visualizing immunoreactive bands, which were imaged using an Amersham Imager 680 (GE Healthcare Life Science). The intensity of the signal was quantified using ImageJ (NIH, Bethesda, Maryland, USA).

### Aβ immunohistochemistry

Paraffin-fixed sagittal brain sections were processed for Aβ immunohistochemistry. Sections were boiled in citrate buffer (pH 6.0, 10 mM trisodium citrate) for 5 min followed by cooling. After blocking with 5% goat serum (S-1000, Vector Laboratories Inc) for 30 min, brain sections were incubated with an anti-82E1 (1 : 100, 10323, IBL) antibody overnight at 4°C followed by incubation with goat anti-mouse Alexa Fluor 488 (A11029, Thermo Fisher Scientific) for 1 h at room temperature. Nuclei were stained with DAPI (P36931, Vector Laboratories Inc). Images were obtained using a microscope (Carl Zeiss). Aβ plaques were evaluated as the percentage of the immunostained area (positive pixels) divided by the total area examined (total pixels) using ImageJ software.

### Immunofluorescence staining and cell proliferation analysis

Immunofluorescence and bromodeoxyuridine (BrdU) staining were conducted using another set of mice. After the probiotic supplementation for four months, the mice (*n* = 6 per each group) were injected with 50 mg of BrdU (027-15561. Wako) per kg body weight for 3 days (twice per day). The mice were anesthetized with sevoflurane, then perfused transcardially with cold PBS and 4% PFA. The brains were quickly removed, postfixed in 4% PFA for 24 h, and then cryoprotected for 2–3 days with 30% sucrose (0.038 M NaH_2_PO_4_ and 0.162 Na_2_HPO_4_). Serial coronal sections were cut on a vibratome (40*μ*m thickness, Leica Microsystems) and collected in a series 12 and stored in a cryoprotection solution (30% sucrose, 150 mM NaCl, and 250 mM polyvinylpyrrolidone in 0.1 M PB) at –20°C until immunofluorescence staining for BrdU, Iba1, and GFAP. For BrdU immunofluorescence staining, the sections were boiled in a citrated buffer on slides in a pressure cooker for 5 min, then washed in PBS. BrdU was stained using a 5-bromo-2-deoxy-uridine labeling and detection kit (11296736001, Roche) in accordance with the manufacturer’s instructions. For the Iba1 and GFAP staining, sections were incubated overnight at 4°C with the appropriate primary antibodies, namely, mouse monoclonal anti-82E1 antibody with rabbit polyclonal anti-Iba1 (1 : 100, 019-19741, Wako) or rabbit polyclonal anti-GFAP (1 : 100, RO1003, Shima Lab) antibodies. The sections were washed and incubated with the secondary antibodies for 1 h, namely, goat anti-mouse Alexa Fluor 488 or 568 and goat anti-rabbit Alexa Fluor 568 antibodies (A11029, A11031, A11036, respectively, ThermoFisher Scientific). Nuclei were stained with DAPI (P36931, Vector Laboratories Inc). Finally, images were obtained using a confocal fluorescence microscope SpinSR10 (Olympus). To quantify the BrdU-positive cells, the number of positive cells in nine sections per each animal was measured. The quantification of Iba1-positive and GFAP-positive cells was performed using ImageJ software in 1×1 mm^2^ square per field in 4 and 2 randomly selected fields in the cortex and hippocampus of each animal, respectively, and the mean was used for statistical analysis.

### Staining of Aβ fibril

We used Thioflavin T (ThT, # SHBN0977, Sigma) for staining Aβ fibril. Brain sections were placed in 0.5% acid alcohol for 5 s, then rinsing in four changes of distilled water. Then the section was stained with 1% ThT for 5 min. Followed by washing well in running water and placing in 1% acetic acid for 15 min. Finally, images were obtained using a confocal fluorescence microscope SpinSR10 (Olympus). To quantify Aβ fibrils; the ThT-positive plaque burdens area was measured using ImageJ software in 4 and 2 randomly selected fields in the cortex and hippocampus, respectively, per each animal. Aβ fibril was evaluated as the percentage of the immunostained area divided by the total area.

### Quantitative real-time PCR (qRT-PCR) analysis

Trizol (Invitrogen) was used to isolate total RNA from the hippocampal and cortical tissues according to the manufacturer’s instructions. The ReverTra Ace qPCR RT Kit (FSQ-101, TOYOBO) was used for cDNA synthesis. qRT-PCR was performed with GeneAce SYBR qPCR Mix (319-07683, Nippon Gene) using the 7500 Fast Real-Time PCR System (Applied Biosystems). All samples were normalized to the corresponding internal gene control, GAPDH gene, following the manufacturer’s instructions. Amplification was performed using these primers: IL-6: sense, 5’-TGATGGATGCTACCAAACTGAT-3’, and antisense, 5’-CTGTGACTCCAGCTTATCTCTTGGT-3’; IL-1β: sense, 5’-GAAGCACCAGCACATTGCTT.

T-3’, and antisense, 5’-GGAGCCTC-ATGGCCCAATTT-3’; TGF-β1 sense, 5’-ACTGGAGTTG-TACGGC-3’, and antisense, 5’-GGGGCTGATCCCGTTG-3’; ADAM10 sense, 5’-CACCAAAAACACCAGCGTGC-3’ and antisense, 5’-AGTGTCCCTCTTCATTCGTAGG-3’; GAPDH: sense, 5’-GCATCTTCTTGTGCAGTGCC-3’, and antisense, 5’-GAGAAGGCAGCCCTGGTAAC-3’.

### Microbiota analysis

All feces were collected at the end of the probiotic supplementation and used for microbiota analysis (probiotic group, *n* = 19 and saline group, *n* = 17). DNA was extracted from 20 mg of feces using GENE PREP STAR PI-480 (Kurabo Industries Ltd., Osaka, Japan) and FastPrep-24 5G Homogenizer (MP Biomedicals, CA, USA). 16S rRNA gene sequencing was performed as previously described [28]. The sequence data obtained were analyzed using QIIME 2 version 2017.10 [29, 30]. Potentially chimeric sequences were removed with DADA2 [31], followed by trimming 30 and 90 bases of the 3’ region of the forward and reverse reads, respectively. The taxonomical classification was conducted using the Naive Bayes classifier trained on the Greengenes13.8 with a 99% threshold of an operational taxonomic unit (OTU) of 16S rRNA full-length sequences. Principal coordinate analysis (PCoA) was carried out on the basis of the weighted UniFrac distance estimated using QIIME 2 software.

### Statistical analysis

Statistical analysis was performed using GraphPad Prism software (GraphPad Software, San Diego, CA). Data are presented as the mean±SD. Student’s *t*-test was used for analyzing statistical significance. A *p*-value < 0.05 was considered significant.

## RESULTS

### B. breve MCC1274 supplementation prevents memory impairment in App^NL-G-F^ mice

We firstly performed a NOR test, which has been previously used to characterize memory impairment in *App^*NL*-*G*-*F*^* mice [32, 33], to determine the effects of oral *B. breve* MCC1274 supplementation on recognition ability and short-term memory in *App^*NL*-*G*-*F*^* mice that exhibit memory and learning deficits at seven months of age [24, 34]. The probiotic and vehicle groups showed similar exploratory preferences between the two familiar objects during the training session (Supplementary Figure 1A), suggesting that both the probiotic and vehicle groups have an equal level of interest toward the familiar objects. However, in the retention session, the vehicle group had no significant difference in exploration time for either the familiar or novel object, whereas the probiotic group had a significantly increased exploration time for the novel object compared with the familiar object (Supplementary Figure 1B). Moreover, the discrimination index (DI) was higher in the probiotics group compared to the vehicle group (Supplementary Figure 1C). This result is consistent with our previous finding that *B. breve* MCC1274 supplementation prevented memory impairment in Aβ-injected mice [15].

### B. breve MCC1274 supplementation reduces Aβ production and deposition in the hippocampus

**Fig. 2:**
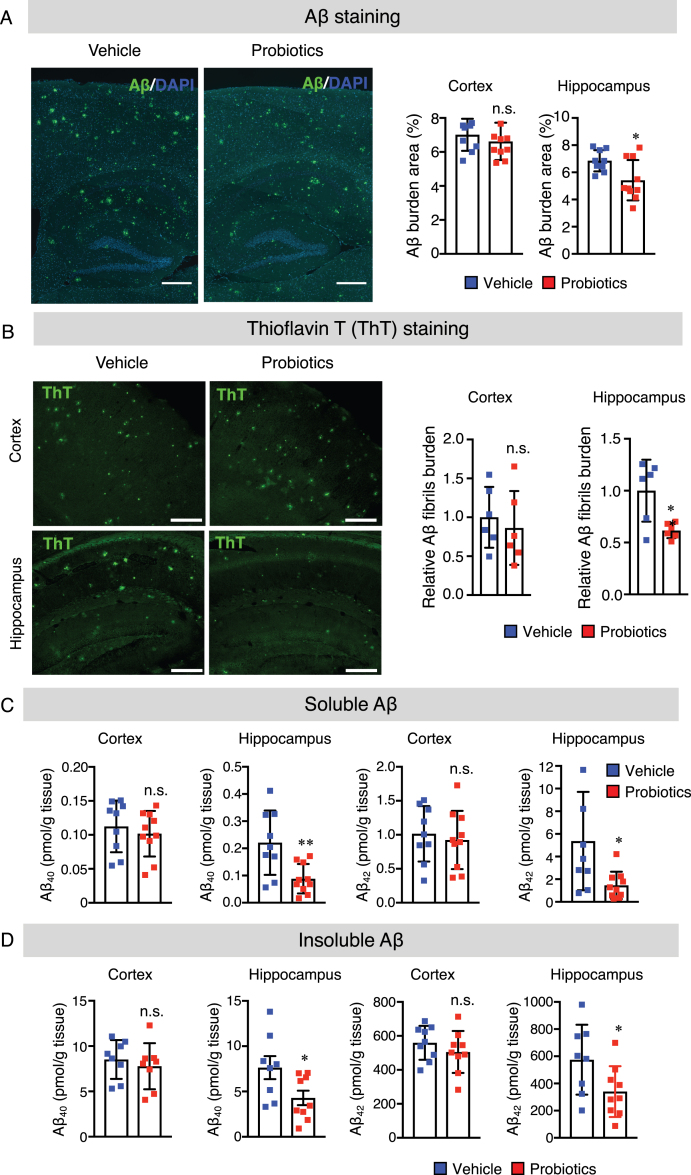
*B. breve* MCC1274 supplementation reduces Aβ plaque burden, Aβ levels, and Aβ fibrils in the hippocampus of *App^*NL*-*G*-*F*^* mice. A) The representative fluorescent images of Aβ plaque burden detected by anti-Aβ antibody (82E1), which recognizes both Aβ_40_ and Aβ_42_ (left panel). Aβ burden areas including the cortex and hippocampus were quantified as the percentage of immunostained area divided by all cortical and hippocampal areas (right panel). Scale bars are 250*μ*m. B) The representative fluorescent images of Aβ fibril were detected by thioflavin T (left panel). Relative Aβ fibril burden in both cortex and hippocampus were quantified (right panel), Scale bars are 100*μ*m. Sandwich ELISA result of cortical and hippocampal levels of soluble Aβ_40_ and Aβ_42_ (C) and insoluble Aβ_40_ and Aβ_42_ (D) of *App^*NL*-*G*-*F*^* mice. Aβ levels were normalized to each tissue weight. Data are expressed as the mean±SD, *n* = 9-10, ^*^p < 0.05, ^**^p < 0.01 compared with the vehicle group, as determined by Student’s *t*-test.

Aβ deposition in the brain is a hallmark of AD development. Therefore, we examined Aβ levels in the brains of mice to evaluate whether the preventing of memory impairment has resulted from the decrease in Aβ levels. We performed immunohistochemical staining for Aβ to evaluate Aβ deposition in the brains. Paraffin-fixed sagittal brain sections were stained with the anti-Aβ antibody (82E1) that recognizes both Aβ_40_ and Aβ_42_. The result demonstrated a significant reduction in the level of Aβ deposition in the hippocampus, but not in the cortex, of the probiotics group compared with the vehicle group (Fig. 2A). Next, we also performed Thioflavin T (ThT) fluorescence staining which is used to quantify the formation of Aβ fibrils to examine whether *B. breve* MCC1274 supplementation suppresses Aβ fibril formation. We found that the percentage of ThT-positive plaque burdens area was significantly lower in the hippocampus of the probiotics group than the vehicle group (Fig. 2B). Also, both hippocampal soluble and insoluble Aβ_40_ and Aβ_42_ levels were significantly lower in the probiotics group than in the vehicle group (Fig. 2C, D). On the contrary, there was no significant difference in the cortical Aβ deposition, Aβ fibril formation as well as soluble and insoluble Aβ_40_ and Aβ_42_ levels between the probiotic and vehicle groups (Fig. 2A-D). Taken together, our findings suggest that *B. breve* MCC1274 supplementation reduces Aβ production, deposition, and fibril formation in the hippocampus. Based on these observations, we propose that the decrease in Aβ levels in the hippocampus by *B. breve* MCC1274 supplementation may prevent the memory impairment seen in *App^*NL*-*G*-*F*^* mice since the hippocampus is the primary area of the brain responsible for memory and learning.

### B. breve MCC1274 supplementation enhances the hippocampal protein level of ADAM10 in App^*NL*-*G*-*F*^ mice

**Fig. 3:**
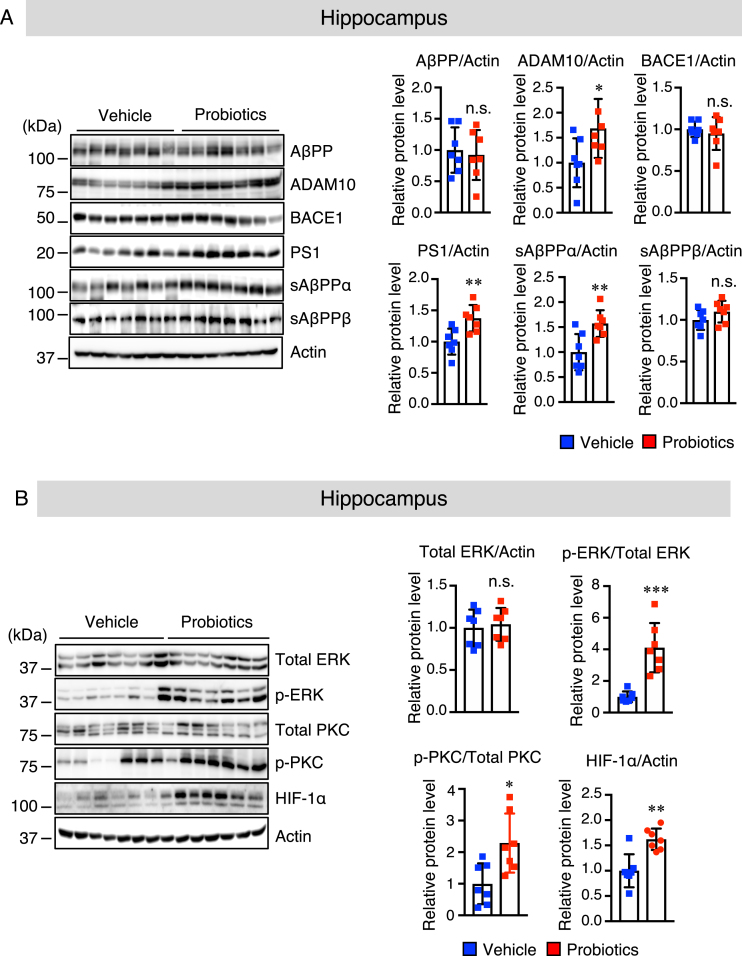
*B. breve* MCC1274 supplementation upregulates ADAM10 protein level in the hippocampus of *App^*NL*-*G*-*F*^* mice. Protein levels of AβPP, ADAM10, BACE1, PS1, sAβPP*α*, sAβPPβ, and actin (A) and protein levels of total ERK, phosphorylated (p) ERK, total PKC, pPKC, HIF-1*α*, and actin (B) in the hippocampus were determined by western blot analysis, quantified by densitometry, normalized to actin level, and expressed as a value relative to the control. Data are expressed as the mean±SD, *n* = 7. ^*^p < 0.05, ^**^p < 0.01, ^***^p < 0.001 compared with the vehicle group, n.s., no significant difference, as determined by Student’s *t*-test.

To provide further insights into the mechanisms by which *B. breve* MCC1274 supplementation decreased Aβ production in the hippocampus, we measured the protein levels of AβPP and its cleavage enzymes, namely: ADAM10 (*α*-secretase), PS1 (a component of *γ*-secretase), and BACE1 (β-secretase) in the hippocampus and cortex of mouse brains by western blotting. The results revealed that *B. breve* MCC1274 supplementation significantly upregulated ADAM10 and PS1 in the hippocampus, whereas AβPP and BACE1 levels did not change (Fig. 3A). However, in the cortex, there was no significant difference in the levels of AβPP, ADAM10, BACE1, and PS1 between the two groups (Supplementary Figure 2A). We further evaluated the protein levels of cortical and hippocampal AβPP fragments, including soluble (s) AβPP*α* and sAβPPβ by western blotting. AβPP can be cleaved by ADAM10 to generate sAβPP*α*. As expected, sAβPP*α* levels in the hippocampus were significantly higher in the probiotic group than in the vehicle group (Fig. 3A), whereas there was no significant difference in the cortex between the two groups (Supplementary Figure 2A). Also, both the cortical and hippocampal sAβPPβ levels were not significantly different between the two groups (Fig. 3A and Supplementary Figure 2A). These findings indicate that *B. breve* MCC1274 supplementation inhibits Aβ production in the hippocampus through the increase in ADAM10.

### B. breve MCC1274 supplementation improves post-transcriptional regulation of ADAM10

It has been reported that hypoxia-inducible factor (HIF)-1*α*-binding sites are essential for the transcriptional activity of the ADAM10 promoter [35]. We sought to determine whether *B. breve* MCC1274 supplementation increases the ADAM10 protein level via HIF-1*α*. We found that the hippocampal HIF-1*α* protein level (Fig. 3B), but not the cortical HIF-1*α* protein level (Supplementary Figure 2B), was significantly higher in the probiotic group than in the vehicle group. Since the MEK/ERK signaling pathway is a key regulator of HIF-1*α* [36], we further assessed whether *B. breve* MCC1274 supplementation could modulate ERK activation. Cortical and hippocampal ERK phosphorylation was assessed by western blotting. Although the total ERK level was unchanged in both the hippocampus (Fig. 3B) and cortex (Supplementary Figure 2B) between the two groups, the phosphorylated (p-) ERK level in the hippocampus (Fig. 3B), but not in the cortex (Supplementary Figure 2B), was higher in the probiotic group than in the vehicle group. Since protein kinase C (PKC) plays a significant role in ERK phosphorylation [37], we, therefore, assessed the cortical and hippocampal levels of p-PKC by western blotting. The level of hippocampal p-PKC was significantly higher in the probiotic group than in the vehicle group (Fig. 3B), whereas there were no significant differences in cortical p-PKC level between the two groups (Supplementary Figure 2B). These findings suggest that *B. breve* MCC1274 supplementation increases the HIF-1*α* level through the PKC-ERK pathway, consequently inducing the upregulation of the ADAM10. Next, to assess if *B. breve* MCC1274 supplementation altered transcription of ADAM10, we assayed the mRNA expression of ADAM10 by qRT-PCR. Although the ADAM10 mRNA expression level was not statistically significant, it tended to be increased in the hippocampus of the probiotic group compared with that of the vehicle group (Supplementary Figure 3), indicating that some unknown factor(s) might be required for the transcription of ADAM10. Therefore, we suggest that the effect of *B. breve* MCC1274 on the increased ADAM10 protein level is likely to be at the post-transcription level, including its stabilization.

### B. breve MCC1274 supplementation does not alter the phosphorylation of tau

**Fig. 4:**
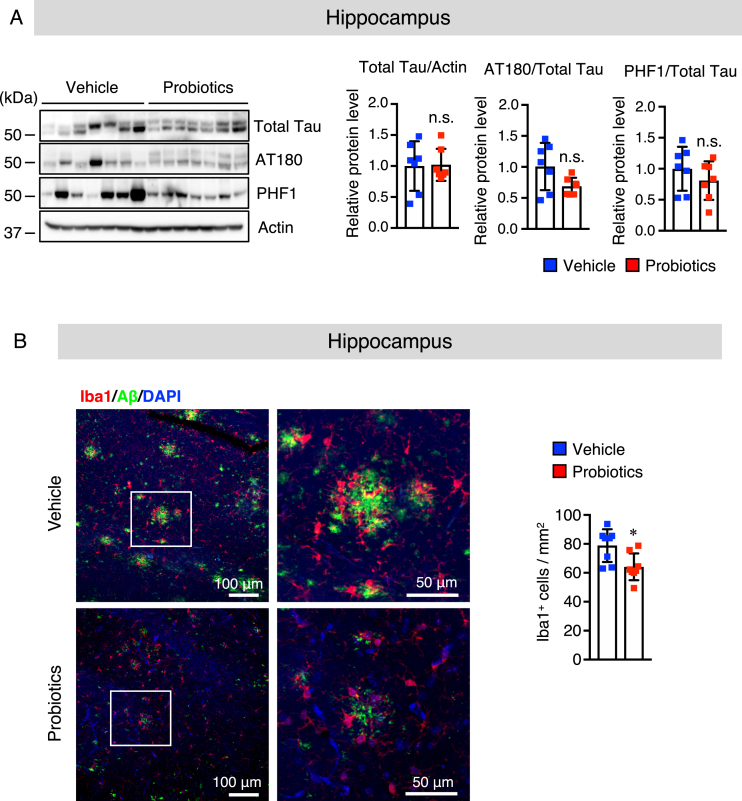
*B. breve* MCC1274 supplementation does not affect the phosphorylation of tau and attenuates microglial activation in the hippocampus of *App^*NL*-*G*-*F*^* mice. A) The protein levels of total Tau, AT180 (p-Thr23), PHF1 (p-Ser369/Ser404), and actin in the hippocampus were determined by western blot analysis, quantified by densitometry, normalized to actin level, and expressed as a value relative to the control. B) Brain sections were stained with the anti-Iba1 (red) and anti-Aβ (green) antibodies, and cell nuclei were stained with DAPI (blue). Representative images of the hippocampus (left panel). Highly magnified images of the squared region in the left panels are shown in the adjacent right panels. Numbers of Iba1-positive cells (right panel) in the hippocampus. Data are expressed as the mean±SD, *n* = 6-7, ^*^p < 0.05 compared with the vehicle group, n.s., no significant difference as determined by Student’s *t*-test.

The tau hyperphosphorylation is a distinctive pathological feature in the brain of AD patients. Excessive tau phosphorylation at Thr23 (AT-180) and Ser396/Ser404 (PHF1) has been found in AD patients’ brains, which inhibits physiological tau binding to microtubules, resulting in memory impairment [38]. Thus, we evaluated whether *B. breve* MCC1274 supplementation can affect tau phosphorylation. There was no significant alteration in total tau, AT-180 (p-Thr23), and PHF1 (p-Ser396/Ser404) levels in both the hippocampus (Fig. 4A) and cortex (Supplementary Figure 4A) between the two groups as determined by western blotting. These findings indicate that tau phosphorylation was not involved in improving cognitive function after *B. breve* MCC1274 supplementation.

### B. breve MCC1274 supplementation attenuates microglial activation in the hippocampus

**Fig. 5:**
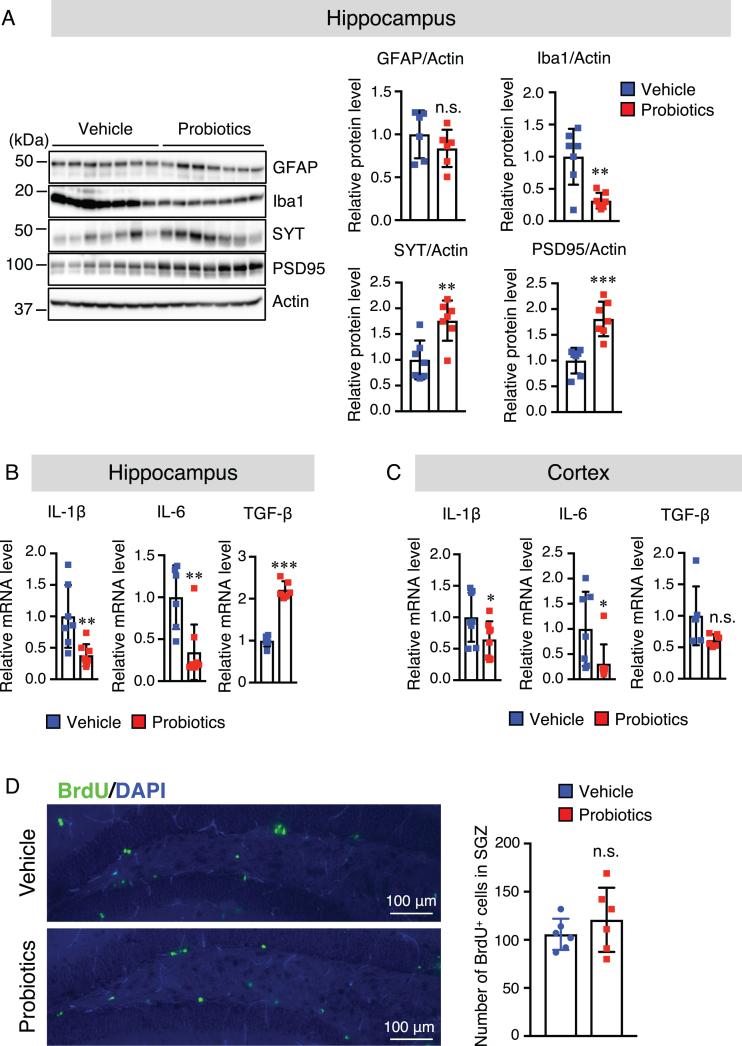
*B. breve* MCC1274 supplementation decreases the Iba1 protein level and increases synaptic protein levels in the hippocampus, whereas it has no effect on cell proliferation in the DG of the hippocampus. A) Protein levels of GFAP, Iba1, SYT, PSD95, and actin in the hippocampus were determined by western blot analysis and quantified by densitometry, normalized to actin level, and expressed as a value relative to the control. IL-6 and IL-1β, and TFG-β1 mRNA expression levels in the hippocampus (B) and cortex (C) were assessed by qRT-PCR analysis. Each mRNA expression was normalized to the corresponding amount of GAPDH mRNA. D) Brain sections were stained with the anti-BrdU antibody (green) and cell nuclei were stained with DAPI (blue) in DG in the hippocampus (left panel). The number of BrdU^+^ cells in the SGZ of DG (right panel). Data are expressed as the mean±SD, *n* = 6-7, ^*^p < 0.05, ^**^p < 0.01, ^***^p < 0.001 compared with the vehicle group n.s., no significant difference as determined by Student’s *t*-test.

Our previous study suggested that oral supplementation of *B. breve* MCC1274 reduced inflammatory gene expression in the hippocampus [15]. However, its effect on glial activation in the brain was not explored in that study. Thus, we investigated whether oral supplementation of *B. breve* MCC1274 can alter glial activation. Brain sections were stained with the anti-Aβ with either the anti-GFAP (an astrocytic marker) or anti-Iba1 (a microglia marker) antibodies. The number of hippocampal Iba1-positive cells was significantly lower in the probiotic group than in the vehicle group (Fig. 4B), whereas, in the cortex, there was no significant difference between both groups (Supplementary Figure 4B). However, in both the hippocampus and cortex, there were no significant differences in the GFAP-positive cell number between both groups (Supplementary Figure 5). To confirm these results, we further assessed GFAP and Iba1 protein levels by western blotting, and similar results were obtained; the hippocampal Iba1 protein level was significantly lower in the probiotic group than in the vehicle group (Fig. 5A), whereas there were no significant differences in the Iba1 protein level in the cortex between both groups (Supplementary Figure 6). The GFAP level was also similar in both the hippocampus and cortex between both groups (Fig. 5A and Supplementary Figure 6). We further investigated the mRNA expression levels of the proinflammatory (IL-1β and IL-6) and anti-inflammatory (TGF-β1) cytokines that are produced by microglia and astrocytes, in the hippocampal and cortical tissues by qRT-PCR analysis. Interestingly, the mRNA expression levels of IL-1β and IL-6 in both the hippocampus and cortex were significantly lower in the probiotic group than in the vehicle group and TGF-β1 mRNA expression level in the hippocampus, but not in the cortex, was higher in the probiotic group than in the vehicle group, (Fig. 5B, C). These findings indicate that aside from microglial activation, other unknown factors altered by *B. brev*e MCC1274 supplementation may be involved in the decrease in cortical IL-6 and IL-1β expression levels. Taken together, these findings suggest that *B. breve* MCC1274 supplementation has an anti-inflammatory function by attenuating microglial activation, and thereby decreasing the expression levels of pro-inflammatory cytokines as well as increasing the expression levels of anti-inflammatory cytokine.

### B. breve MCC1274 supplementation increases the synaptic protein levels in the hippocampus

We also evaluated the effect of *B. breve* MCC1274 supplementation on synapses in the hippocampus and cortex using the anti-synaptotagmin (SYT, presynaptic protein) and anti-PSD95 (postsynaptic protein) antibodies by western blot analysis. The results of this analysis showed that the protein levels of SYT and PSD95 in the hippocampus were significantly higher in the probiotic group than in the vehicle group (Fig. 5A), whereas there were no significant differences in their protein levels in the cortex between both groups (Supplementary Figure 6). These findings suggest that *B. breve* MCC1274 supplementation increases synaptic density in the hippocampus.

### B. breve MCC1274 supplementation does not affect cell proliferation in the subgranular zone of the dentate gyrus

To examine the effect of *B. breve* MCC1274 supplementation on the cell proliferation in the subgranular zone (SGZ) of the dentate gyrus (DG) of the hippocampus, *App^*NL*-*G*-*F*^* mice were injected with BrdU. The number of BrdU-positive cells was calculated in this area for both the probiotic and vehicle groups. Although the difference in the BrdU-positive cell number in the SGZ of the DG area was not statistically significant, they tended to be higher in the probiotic group than in the vehicle group (Fig. 5D).

### B. breve MCC1274 supplementation does not change gut microbiota composition

**Fig. 6:**
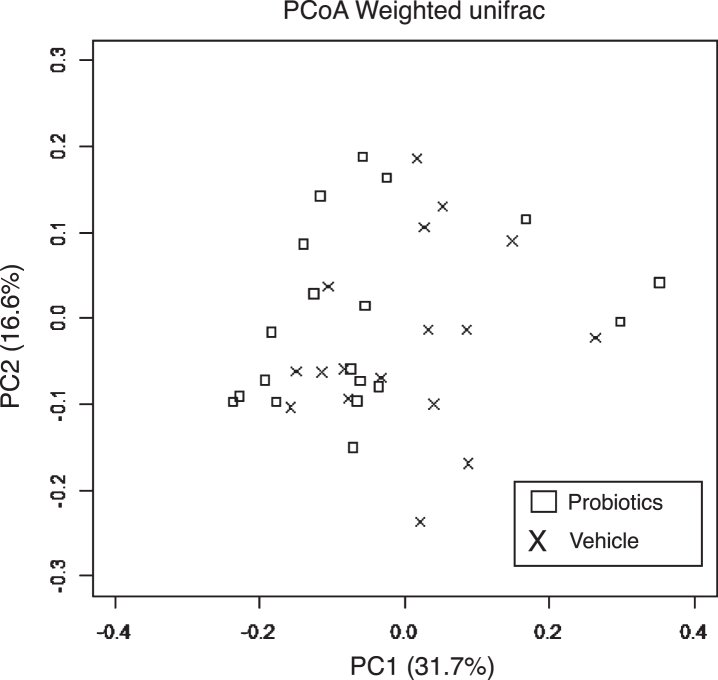
Effects of *B. breve* MCC1274 supplementation on gut microbiota. PCoA based on weighted unifrac distance from 16S rRNA sequencing data did not show a significant difference between *App^*NL*-*G*-*F*^* mice with and without *B. breve* MCC1274 supplementation as determined by permutation MANOVA (*p* =0.379).

Fecal samples were analyzed to investigate whether *B. breve* MCC1274 supplementation caused any changes in the gut microbiota composition. PCoA based on weighted unifrac distance showed no intergroup difference (Fig. 6). Furthermore, in the analysis at the genus level, no change in the gut microbiota composition caused by the administration of probiotics was observed (Table 1). These results are consistent with our previous finding that *B. breve* MCC1274 supplementation does not affect gut microbiota in ddY mice induced by intracerebroventricular injection with Aβ [15].

**Table 1:** Effects of *B. breve* MCC1274 supplementation on gut microbiota at the genus level. There was no significant difference in composition of the gut microbiota at the genus level between *App^*NL*-*G*-*F*^* mice with and without *B. breve* MCC1274 supplementation as determined by Welch’s *t*-test

						Probiotics (%)	Vehicle (%)	*p*
k_Bacteria	p_Actinobacteria	c_Actinobacteria	o_Bifidobacteriales	f_Bifidobacteriaceae	g_Bifidobacterium	0.35	0.78	0.212
k_Bacteria	p_Bacteroidetes	c_Bacteroidia	o_Bacteroidales	f_Bacteroidaceae	g_Bacteroides	2.32	2.32	0.997
k_Bacteria	p_Bacteroidetes	c_Bacteroidia	o_Bacteroidales	f_Porphyromonadaceae	g_Parabacteroides	0.56	0.58	0.903
k_Bacteria	p_Bacteroidetes	c_Bacteroidia	o_Bacteroidales	f_Prevotellaceae	g_Prevotella	2.91	2.36	0.485
k_Bacteria	p_Bacteroidetes	c_Bacteroidia	o_Bacteroidales	f_Rikenellaceae	g_	1.40	1.55	0.650
k_Bacteria	p_Bacteroidetes	c_Bacteroidia	o_Bacteroidales	f_S24-7	g_	34.23	31.71	0.490
k_Bacteria	p_Bacteroidetes	c_Bacteroidia	o_Bacteroidales	f_[Paraprevotellaceae]	g_[Prevotella]	7.81	7.48	0.870
k_Bacteria	p_Firmicutes	c_Bacilli	o_Lactobacillales	f_Lactobacillaceae	g_Lactobacillus	10.51	13.99	0.339
k_Bacteria	p_Firmicutes	c_Bacilli	o_Turicibacterales	f_Turicibacteraceae	g_Turicibacter	4.12	5.13	0.656
k_Bacteria	p_Firmicutes	c_Clostridia	o_Clostridiales	_	_	3.35	4.20	0.444
k_Bacteria	p_Firmicutes	c_Clostridia	o_Clostridiales	f_	g_	4.49	3.43	0.304
k_Bacteria	p_Firmicutes	c_Clostridia	o_Clostridiales	f_Lachnospiraceae	_	9.58	6.51	0.270
k_Bacteria	p_Firmicutes	c_Clostridia	o_Clostridiales	f_Lachnospiraceae	g_	0.62	0.75	0.710
k_Bacteria	p_Firmicutes	c_Clostridia	o_Clostridiales	f_Lachnospiraceae	g_Coprococcus	2.01	1.54	0.328
k_Bacteria	p_Firmicutes	c_Clostridia	o_Clostridiales	f_Lachnospiraceae	g_[Ruminococcus]	0.78	0.53	0.245
k_Bacteria	p_Firmicutes	c_Clostridia	o_Clostridiales	f_Ruminococcaceae	g_Oscillospira	1.26	0.75	0.060
k_Bacteria	p_Firmicutes	c_Clostridia	o_Clostridiales	f_Ruminococcaceae	g_Ruminococcus	1.73	1.25	0.170
k_Bacteria	p_Firmicutes	c_Erysipelotrichi	o_Erysipelotrichales	f_Erysipelotrichaceae	g_Allobaculum	5.07	8.24	0.288

## DISCUSSION

Several studies provided strong evidence for the link between the gut microbiota and the brain through the microbiota-gut-brain axis. Therefore, any disruption or alteration in an individual’s gut microbiota can affect the individual’s behavior and cause psychiatric and neurodegenerative diseases, including AD. Recently, we have demonstrated that orally administered *B. breve* A1 (MCC1274) prevented cognitive deficits in Aβ-injected mice through the suppression of hippocampal inflammation and immunoreactive genes [15] and that providing the same probiotic to MCI subjects for 16 weeks improved their cognitive function [23]. However, the underlying molecular mechanism by which probiotic supplementation prevents cognitive deficits in humans and animals has not been fully elucidated. In this study, therefore, we further investigated the role of *B. breve* MCC1274 in AD-like pathologies to evaluate its ability to alter Aβ production and glial activation in *App^*NL*-*G*-*F*^* mice. Here, we demonstrated that *B. breve* MCC1274 supplementation significantly prevented memory impairment and found decreased soluble and insoluble Aβ levels as well as the Aβ plaque load in the hippocampus. We also revealed that *B. breve* MCC1274 supplementation attenuated microglial activation accompanied by the reduction in IL-1β and IL-6 mRNA expression levels as well as the increases in TGF-β1 mRNA expression levels and synaptic protein levels.

Several transgenic mouse models overexpressing human AβPP have been generated for use in the investigation of AD-like pathologies and drug treatments for AD. However, these mouse models overexpress AβPP and AβPP fragments such as sAβPP, CTF*α*, and CTFβ, which may possess some biological functions and cause the expression of artefactual phenotypes. Recently, *App^*NL*-*G*-*F*^* mice carrying Swedish, Beyreuther/Iberian, and Arctic mutations associated with familial AD have been generated to overcome these problems. Since these mice showed cognitive impairment, Aβ amyloidosis, and neuroinflammation without physiological AβPP overexpression, we, therefore, used this mouse model in this study.

It has been demonstrated that the supplementation of some probiotics such as *Bifidobacterium longum* and *Lactobacillus acidophilus* significantly improved the cognitive function in Aβ-treated rats and AβPP/PS1 transgenic mice [39, 40]. We also previously demonstrated that *B. breve* MCC1274 supplementation ameliorated the cognitive decline in Aβ-injected mice [15] and improved the cognitive function in MCI subjects [23]. In this study, we conducted the NOR test to evaluate short-term memory and recognition ability and found that *App^*NL*-*G*-*F*^* mice treated with saline showed short-term memory impairment at the age of seven months, which is consistent with a previous report [24]. However, the mice supplemented with *B. breve* MCC1274 did not show memory impairment, suggesting that *B. breve* MCC1274 supplementation might prevent cognitive impairment in AD.

There is strong evidence showing that the accumulation of Aβ in the brain can induce memory impairment. Therefore, reducing Aβ levels and thereby preventing Aβ toxicity in the brain are thought to be preventive therapy for AD progression. It has been reported that some probiotics such as *Bifidobacterium longum, Lactobacillus acidophilus,* and *L. plantarum* supplemented with memantine decreased the Aβ levels in the brain of AβPP/PS1 transgenic mice [40, 41]. Here, we also found that *B. breve* MCC1274 supplementation significantly decreased hippocampal Aβ production and Aβ plaque load of *App^*NL*-*G*-*F*^* mice, indicating that this probiotic may protect the hippocampus against the toxic effect of Aβ and thus prevent short-term memory impairment.

Although some probiotics can reduce Aβ levels in the brain, the precise mechanism by which these probiotics decrease Aβ levels has not been completely clarified. Here, we found that *B. breve* MCC1274 supplementation increased the hippocampal ADAM10 protein level associated with the increased sAβPP*α* and CTF*α* levels, leading to decreased Aβ levels in the hippocampus. Thus, our findings suggest that *B. breve* MCC1274 supplementation may contribute to AβPP metabolism toward the non-amyloidogenic pathway through the upregulation of the hippocampal ADAM10 protein level, resulting in the decrease in hippocampal Aβ production and deposition.

The PKC-ERK-HIF-1*α* signaling pathway has been involved in ADAM10 transcription. Here, we found that although *B. breve* MCC1274 supplementation activated PKC and ERK leading to the increased ADAM10 protein level, we observed no increase in the ADAM10 mRNA expression level, indicating that some unknown factor(s) might be required for the transcription of ADAM10. Therefore, we suggest that the effect of *B. breve* MCC1274 on the increased ADAM10 protein level is likely to be at the post-transcription level, including its stabilization. Further investigation is required to explore which factor(s) are involved in ADAM10 transcription.

Since previous studies have shown that the improvement of synaptic activity increased ADAM10 levels [42, 43], the increase in synaptic density by *B. breve* MCC1274 supplementation might also be involved in the increase in ADAM10 level. Furthermore, the enhancement of ERK activation, particularly in the hippocampus, is necessary for the increase in synaptic protein levels and memory formation [44]. Here, we found that *B. breve* MCC1274 supplementation markedly increased both presynaptic (SYT) and postsynaptic (PSD95) protein levels and enhanced ERK activation in the hippocampus. Taken together, the enhancement of ERK activation by *B. breve* MCC1274 supplementation may play a role in the increase in synaptic density and subsequently an increase in the ADAM10 level as well as memory formation.

The effect of *B. breve* MCC1274 on tau phosphorylation is unknown. Thus, we also examined whether *B. breve* MCC1274 supplementation can affect tau phosphorylation. Hyperphosphorylated tau at AT180 and PHF1 epitopes has been indicated to suppress its binding to microtubules, thereby diminishing its function [38]. Our results demonstrated that *B. breve* MCC1274 supplementation could not alter tau phosphorylation at AT180 and PHF1 epitopes in both the cortex and hippocampus. Therefore, the improvement of brain performance induced by *B. breve* MCC1274 supplementation is not associated with tau phosphorylation.

Activated microglia and astrocytes are considered pathological hallmarks of AD as they induce both Aβ deposition and release of proinflammatory cytokines such as TNF*α*, IL-1β, and IL-6 [5, 45, 46]. It has been shown that IL-1β and IL-6 play roles in regulating cognitive function; blocking IL-1β in AD mouse models protects against cognitive deficits [47], and the upregulation of IL-6 contributes to cognitive deficits in humans [48]. Several studies have shown that some probiotic strains appeared to have an anti-inflammatory effect, which effectively attenuated cognitive dysfunction [49, 50]. We also previously demonstrated that oral *B. breve* MCC1274 supplementation suppressed the inflammation in the hippocampus of Aβ-injected mice. Here, we demonstrated that *B. breve* MCC1274 supplementation significantly decreased both the number of Iba1^+^ cells surrounding Aβ plaques and the Iba1 protein level in the hippocampus of *App^*NL*-*G*-*F*^* mice. It has been reported that there are two major phenotypes of activated microglia. M1 and M2, which secrete pro-inflammatory and anti-inflammatory cytokines, respectively. In this study, *B. breve* MCC1274 supplementation decreased the mRNA expression levels of pro-inflammatory cytokines (IL-6 and IL-1β) and increased that of an anti-inflammatory cytokine (TGF-β1) in the hippocampus. These results suggest that *B. breve* MCC1274 supplementation might convert M1 to M2 phenotype and that the altered levels of pro- and anti-inflammatory cytokines could be derived from microglia because the number of GFAP^+^ cells and GFAP protein levels were not altered by *B. breve* MCC1274 supplementation. Taken together, our results suggest that *B. breve* MCC1274 supplementation may shift activated microglia from a pro-inflammatory M1 to an anti-inflammatory M2 phenotype. Further investigation is required to test this hypothesis using specific microglia phenotype markers.

Our findings showed that IL-6 and IL-1β mRNA expression levels were decreased in both the cortex and hippocampus of mice given *B. breve* MCC1274 supplementation; the reduction in proinflammatory cytokine expression levels in the hippocampus may be due to the attenuation of microglia activation, whereas that in the cortex may be a result of the reduction of systemic anti-inflammatory cytokine release. Previous animal and human studies showed that probiotics have an intestinal anti-inflammatory function that leads to the reduction in intestinal IL-1β and IL-6 levels [51–53]. Moreover, ingested probiotics plays a systemic anti-inflammatory role and decrease the levels of proinflammatory cytokines released in serum, such as IL-6 and IL-1β [54]. Thus, *B. breve* MCC1274 supplementation might reduce the inflammatory reaction in the brain of *App^*NL*-*G*-*F*^* mice, at least in part, by attenuating microglial activation.

Interestingly, the effects of *B. breve* MCC1274 on Aβ pathology, microglial activation, and synaptic protein levels were found only in the hippocampus and not in the cortex in this study. Although the reason for this is unclear, one possibility is that probiotics can activate specific brain regions through the vagus afferent nerve that is involved in the communication of the microbiome-gut-brain axis [55]. For example, the ingestion of a *Lactobacillus* strain upregulated the mRNA expression of GABA receptors in a regional-dependent manner through the vagus nerve, which could be stimulated by the probiotics. Further studies are necessary to elucidate the potential mechanisms associated with the effects of *B. breve* MCC1274 on the hippocampus.

Reduction of hippocampal neurogenesis has been reported in AD cases of both human and AD-like mouse models. A previous study showed that *L. helveticus* R0052 and *B. longum* R0175 prevent the alteration of neurogenesis caused by chronic stress [56], and it has been reported that VSL#3 can reverse the impairment of neurogenesis that occurred as a result of antibiotics treatment [57]. Therefore, we examined whether *B. breve* MCC1274 supplementation can affect cell proliferation in the hippocampus of *App^*NL*-*G*-*F*^* mice. In this study, we found that BrdU^+^ cell number did not significantly differ between the probiotic and vehicle groups. The difference between our findings and the previous findings may be due to the differences in the probiotic strain and mouse type used. Thus, *B. breve* MCC1274 supplementation is not effective in improving hippocampal neurogenesis in *App^*NL*-*G*-*F*^* mice.

In this study, we did not observe a marked change in the gut microbiota following the administration of *B. breve* MCC1274, which is consistent with our previous finding [15], implying that another mechanism(s) might exist for ameliorating AD-like pathologies. Our previous study has shown that viable or nonviable *B. breve* MCC1274 could also prevent cognitive impairment and suppress inflammatory gene expression in the hippocampus of AD-like mice, implying that some structural components of the probiotics may be involved in the modulation of neuronal immune responses [15]. Thus, one possible mechanism by which the probiotics ameliorate AD-like pathologies is that some structural components are likely to be involved in their effects. However, further investigation on whether nonviable *B. breve* MCC1274 has ameliorating effects on AD-like pathologies is necessary.

In summary, we have shown that oral *B. breve* MCC1274 supplementation alleviated cognitive dysfunction and Aβ production and deposition in *App^*NL*-*G*-*F*^* mice. More importantly, we showed for the first time that oral supplementation of *B. breve* MCC1274 positively regulated the ADAM10 protein level in the hippocampus leading to the reduction of Aβ production. The oral *B. breve* MCC1274 supplementation also alleviated neuroinflammation in the brains of *App^*NL*-*G*-*F*^* mice, thereby demonstrating its neuroprotective properties. Our findings support the possibility that oral *B. breve* MCC1274 supplementation might potentially prevent AD progression.

## Supplementary Material

Supplementary Material
