# Supplementary material for: Probiotic Bifidobacterium breve Prevents Memory Impairment Through the Reduction of Both Amyloid-β Production and Microglia Activation in APP Knock-In Mouse
Source: J Alzheimers Dis. 2022 Feb 15;85(4):1555–71. doi: 10.3233/JAD-215025 (PMC8925106; doi:10.3233/JAD-215025)

Supplementary Material

Probiotic *Bifidobacterium breve* prevents memory impairment through the reduction of both Aβ production and microglia activation in APP knock-in mouse

Supplementary Figure 1. *B. breve* MCC1274 supplementation ameliorates memory impairment in $App^{NL\text{-}G\text{-}F}$ mice. Average exploration time for both familiar objects (a and b) during the training (A) and retention (B) session. A) Vehicle- and probiotics-treated mice showed the same exploratory time between the two familiar objects (a, b). B) The vehicle-treated mice spent the same exploratory time between the two familiar objects (a) and the novel object (c). However, the probiotics-treated mice spent a longer time exploring the novel object (c) than the familiar object (a). C) Discrimination index for vehicle and probiotics groups. Data are expressed as the mean ± SD, n = 18 in each group, *$p < 0.05$, compared with the vehicle group, as determined by Student's t-test.

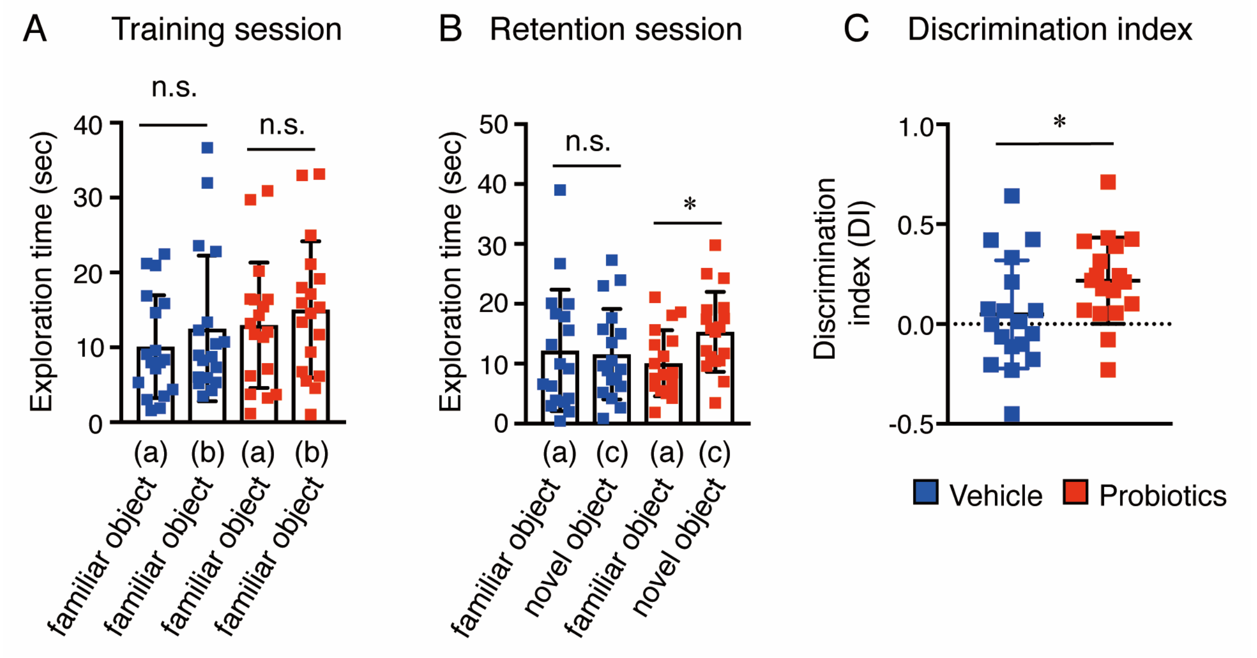

Supplementary Figure 2. *B. breve* MCC1274 supplementation does not affect ADAM10 protein level as well as PKC-ERK-HIF-1α signaling pathway in the cortex of $App^{NL\text{-}G\text{-}F}$ mice. Protein levels of AβPP, ADAM10, BACE1, PS1, sAβPPα, sAβPPβ, and actin (A) and protein levels of total ERK, phosphorylated (p) ERK, total PKC, pPKC, HIF-1α, and actin (B) in the cortex were determined by western blot analysis, quantified by densitometry, normalized to actin level, and expressed as a value relative to the control. Data are expressed as the mean ± SD, n = 7, n.s., no significant difference, as determined by Student's t-test.

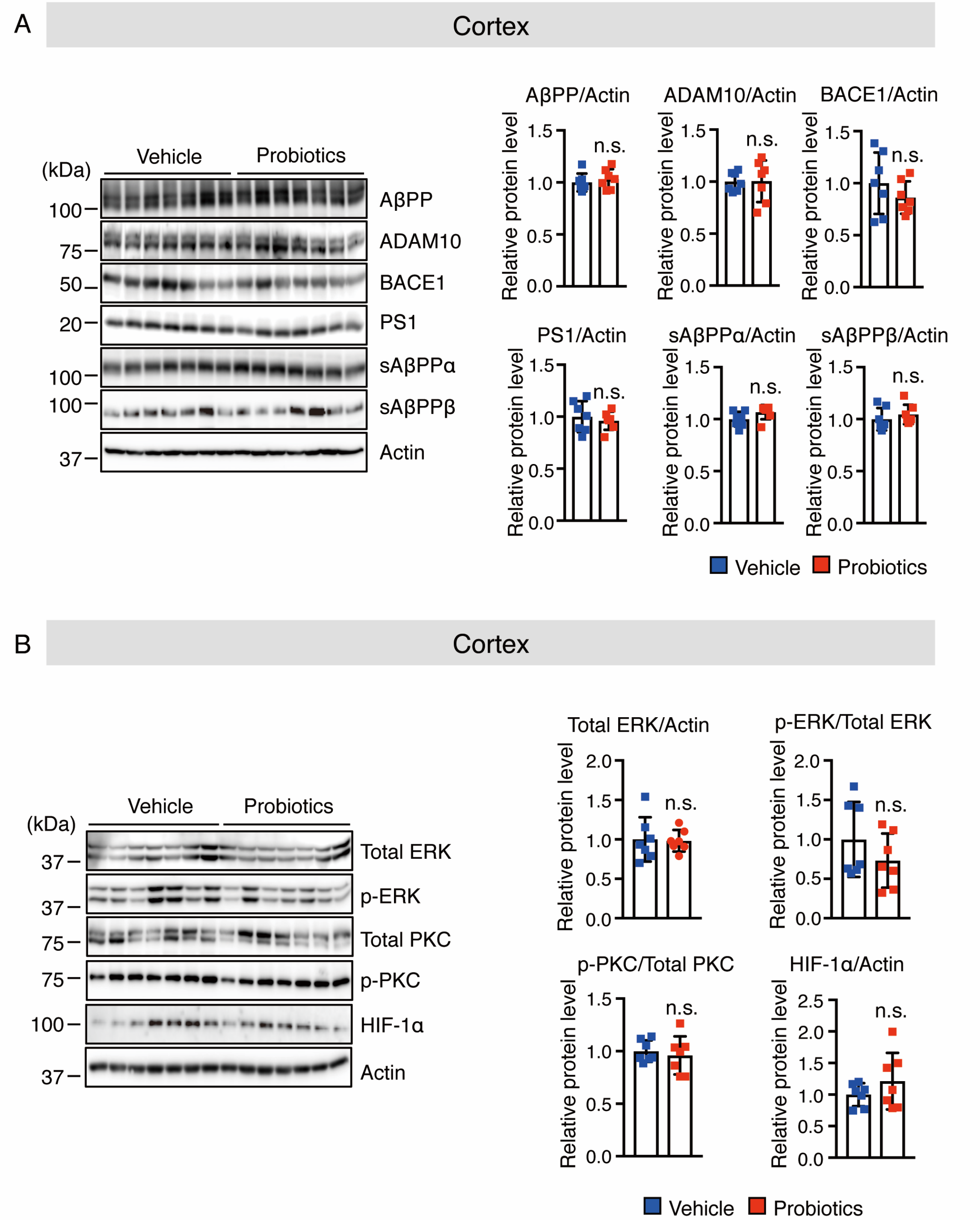

Supplementary Figure 3. *B. breve* MCC1274 supplementation does not affect mRNA expression of ADAM10 in the brain of $App^{NL\text{-}G\text{-}F}$ mice. ADAM10 mRNA expression level in the hippocampus (A) and cortex (B) were assessed by qRT-PCR analysis. The mRNA expression level was normalized to the corresponding amount of GAPDH mRNA. Data are expressed as the mean ± SD, n = 6, n.s., no significant difference, as determined by Student's t-test.

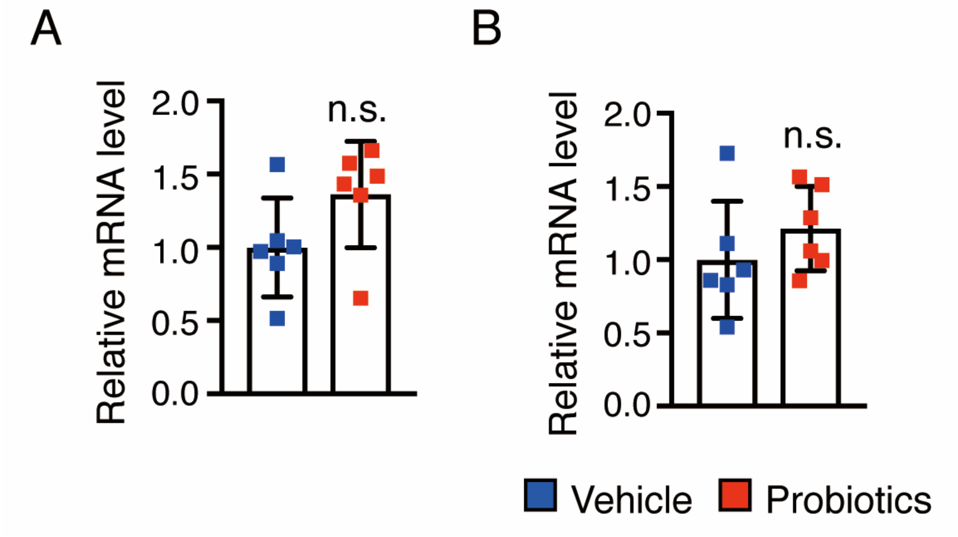

Supplementary Figure 4. *B. breve* MCC1274 supplementation does not affect the phosphorylation of tau and microglial activation in the cortex of *App*$^{NL\text{-}G\text{-}F}$ mice. A) The protein levels of total Tau, AT180 (p-Thr23), and PHF1 (p-Ser369/Ser404) in the cortex were determined by western blot analysis, quantified by densitometry, normalized to actin level, and expressed as a value relative to the control. B) Brain sections were stained with the anti-Iba1 (red) and anti-Aβ (green) antibodies, and cell nuclei were stained with DAPI (blue). Representative images of the cortex (left panel). Highly magnified images of the squared region in the left panels are shown in the adjacent right panels. Numbers of Iba1-positive cells in the cortex (right panel). Data are expressed as the mean ± SD, n = 6-7, n.s., no significant difference as determined by Student's t-test.

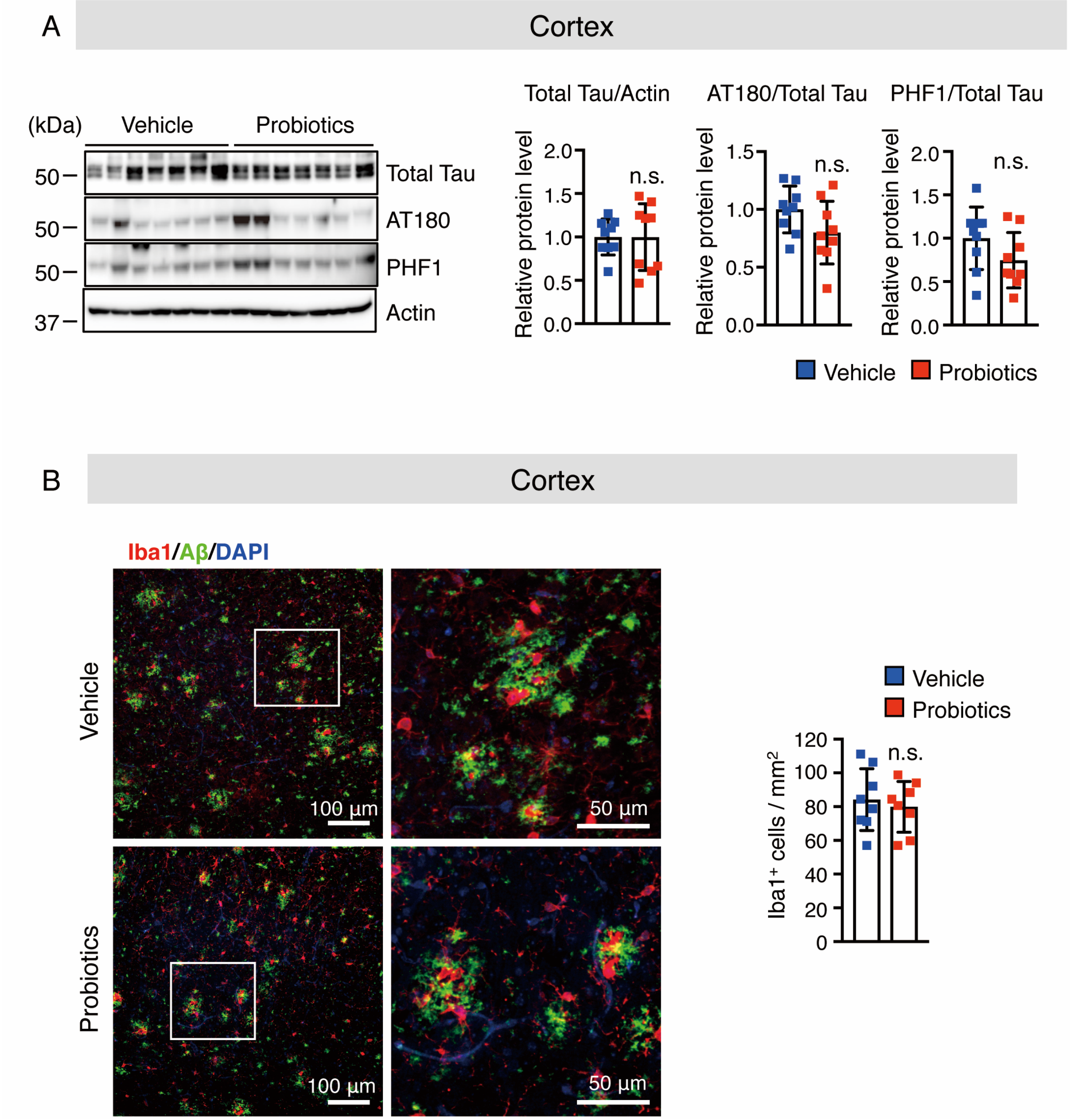

Supplementary Figure 5. *B. breve* MCC1274 supplementation does not affect astrocyte activation in the brain of $App^{NL\text{-}G\text{-}F}$ mice. Brain sections were stained with the anti-GFAP (red) and anti-Aβ (green) antibodies, and cell nuclei were stained with DAPI (blue). Representative images of the hippocampus (A) and cortex (B). Highly magnified images of the squared region in the left panels are shown in the adjacent right panels. Numbers of GFAP-positive cells in the hippocampus (A, right panel) and the cortex (B, right panel). Data are expressed as the mean ± SD, n = 6, n.s., no significant difference as determined by Student's t-test.

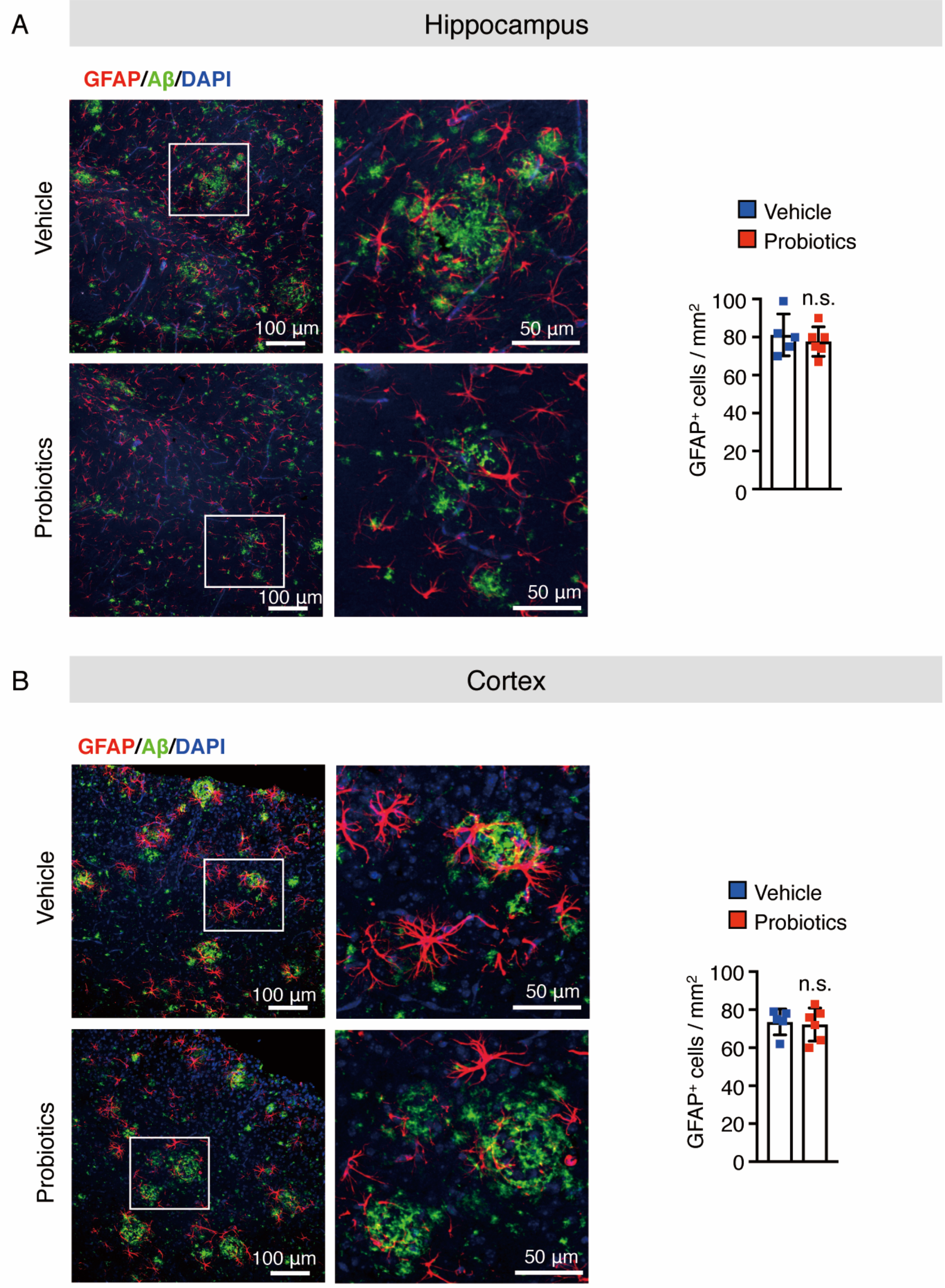

Supplementary Figure 6. *B. breve* MCC1274 supplementation does not affect the protein levels of GFAP, Iba1, SYT, PSD95 in the cortex of $App^{NL\text{-}G\text{-}F}$ mice. Protein levels of GFAP, Iba1, SYT, PSD95, and actin in the cortex were determined by western blot analysis, quantified by densitometry, normalized to actin level, and expressed as a value relative to the control. Data are expressed as the mean ± SD, n = 7, n.s., no significant difference as determined by Student's t-test.

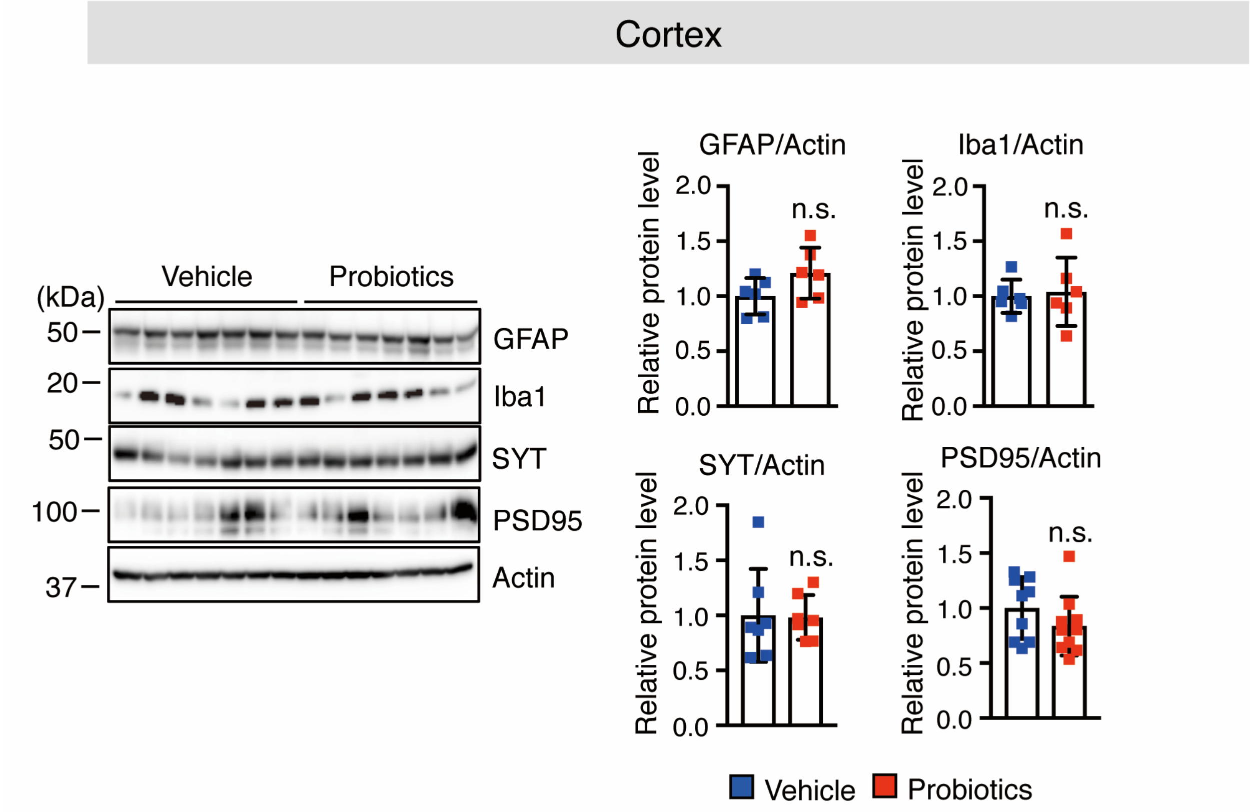

Supplement: Supplementary Material [file jad-85-jad215025-s001.pdf]
